# Coherent control of collective nuclear quantum states via transient magnons

**DOI:** 10.1126/sciadv.abc3991

**Published:** 2021-01-29

**Authors:** Lars Bocklage, Jakob Gollwitzer, Cornelius Strohm, Christian F. Adolff, Kai Schlage, Ilya Sergeev, Olaf Leupold, Hans-Christian Wille, Guido Meier, Ralf Röhlsberger

**Affiliations:** 1 Deutsches Elektronen-Synchrotron DESY, Notkestraße 85, 22607 Hamburg, Germany.; 2 The Hamburg Centre for Ultrafast Imaging, Luruper Chaussee 149, 22761 Hamburg, Germany.; 3 Max-Planck Institute for the Structure and Dynamics of Matter, Luruper Chaussee 149, 22761 Hamburg, Germany.; 4 Friedrich-Schiller Universität Jena, Max-Wien-Platz 1, 07743 Jena, Germany.; 5 Helmholtz-Institut Jena, Fröbelstieg 3, 07743 Jena, Germany.; 6 GSI Helmholtzzentrum für Schwerionenforschung GmbH, Planckstraße 1, 64291 Darmstadt, Germany.

## Abstract

Ultrafast and precise control of quantum systems at x-ray energies involves photons with oscillation periods below 1 as. Coherent dynamic control of quantum systems at these energies is one of the major challenges in hard x-ray quantum optics. Here, we demonstrate that the phase of a quantum system embedded in a solid can be coherently controlled via a quasi-particle with subattosecond accuracy. In particular, we tune the quantum phase of a collectively excited nuclear state via transient magnons with a precision of 1 zs and a timing stability below 50 ys. These small temporal shifts are monitored interferometrically via quantum beats between different hyperfine-split levels. The experiment demonstrates zeptosecond interferometry and shows that transient quasi-particles enable accurate control of quantum systems embedded in condensed matter environments.

## Teaser

A collective nuclear state is coherently controlled via a solid’s quasi-particle with zeptosecond precision.

## INTRODUCTION

Ultraprecise probing and control of processes on their intrinsic time scales are essential to reveal fundamental dynamics in nature. The control of quantum systems is mostly performed via coherent excitation with photons. Coherent light-matter interaction in the hard x-ray regime couples quantum states with electromagnetic waves. The latter exhibit oscillation periods on the order of zeptoseconds associated with the involved x-ray energies of a few kiloelectron volts. Several quantum effects in atoms proceed on these time scales including a host of atomic and nuclear transitions and light-matter interactions like diffraction, Compton scattering, Raman scattering, or resonant x-ray scattering. Although quantum optical concepts have been established in the regime of hard x-rays using nuclear (*1*–*8*) or electronic (*9*) resonances, the associated zeptosecond time scale prevents application of established quantum optical control schemes at x-ray energies. Coherent control of high-energy quantum systems has been achieved by coupling to x-ray cavities (*2*, *3*, *9*), vibration of resonant absorbers (*5*, *7*), magnetic switching (*1*, *4*), or strong coupling of nuclear ensembles (*6*). Precise dynamic control of quantum systems at x-ray energies is at the forefront of contemporary research. Consequently, novel control schemes at these energies are called for to access these time scales and the corresponding phase shifts.

A fascinating opportunity to modify intrinsic quantum properties of solids, like superconductivity, is the excitation of phonons (*10*). The extension of such a scheme to quantum systems embedded in a solid-state host is an attractive approach, which could provide several ways for quantum control via a variety of possible quasi-particles of the vibrational, electronic, or magnetic degrees of freedom in the solid. The embedded quantum systems include electronic or nuclear levels of atoms in the condensed matter state, nitrogen vacancies in diamond (*11*), and ion-doped solids (*12*), among others. With such an additional opportunity to influence the embedded quantum system via quasi-particle excitation, its dynamics may be controlled with high precision. Here, we introduce such a novel method for coherent quantum phase shifts with subattosecond precision. An ensemble of ^57^Fe Mössbauer nuclei embedded in a ferromagnetic film is controlled via transient magnons in the time domain. This quasi-particle control preserves the coherence of the collectively excited nuclear state. Specifically, we use a uniform magnon mode (*13*) to modulate the magnetic hyperfine interactions of the nuclei and to control its quantum phase.

Temporal control of quantum phases yields fundamental knowledge about quantum systems (*14*–*19*) and is a cornerstone of many quantum technologies (*17*, *20*). Nowadays, attosecond electronic processes can be captured in atoms (*21*, *22*), molecules (*23*), or condensed matter (*24*, *25*), leading to the basic understanding of quantum phenomena like tunneling (*26*), ionization of molecules (*27*), or electron scattering (*24*). To achieve this, small time delays, or the corresponding phases, of the system have to be monitored. Currently, time delays shorter than attoseconds are not accessible with laser sources (*28*–*30*), and access to the zeptosecond time delays is still a great challenge (*20*). The understanding and control of quantum phases, however, yield fundamental concepts like the Berry phase (*15*) and the Aharonov-Bohm effect (*19*), both based on the geometric phase. The manipulation of the spectral response of atomic resonances and the study of their ultrafast dynamics are accomplished by the control of the dynamic phase (*16*, *18*). Scaling these concepts toward shorter wavelengths and shorter time scales is indispensable for understanding and controlling high-energy quantum dynamics in general, with potential applications in ultrafast photonic control in a variety of fields ranging from precision interferometry to signal processing in nanoscale photonic devices.

## RESULTS

**Fig. 1:**
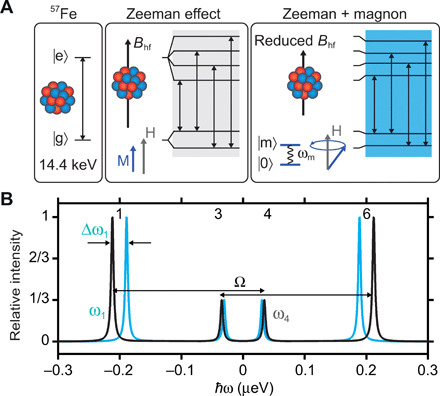
Nuclear-level scheme with magnon excitation. (**A**) Level scheme of a^57^Fe nucleus with the transition from the ground ∣g⟩ to the excited state ∣e⟩ at 14.4 keV. The magnetic hyperfine-field *B*_hf_ splits the nuclear transition by the Zeeman effect. The four circularly polarized transitions are shown that are excitable in our experimental geometry. A static magnetic field H aligns the magnetization M and the hyperfine field in the sample. When a coherent magnon∣m⟩ is excited in the magnetic material at its resonance ω_m_, the magnetization precesses around the external field H. In that case, the hyperfine field is reduced, leading to a shift of the nuclear transitions. (**B**) Energy spectrum of the circularly polarized nuclear transitions in a permalloy film. Black shows the transitions for a static hyperfine field of *B*_hf_ = 27.9 T. Blue shows the shifted transitions for a hyperfine field reduced by 3.0 T as can be induced by magnons. The frequency difference between the transitions with same polarization is Ω.

The concept of quantum phase control with a quasi-particle is based on a temporally well-controlled frequency change of atomic or nuclear transitions. Here, we have chosen the 14.4-keV nuclear transition in ^57^Fe with ω_γ_ = 2.19 × 10^19^ s^−1^, corresponding to an oscillation period of 287 zs. A level scheme of this transition is shown in Fig. 1A. The nuclei are embedded in a ferromagnetic thin film, and the internal magnetic hyperfine field *B*_hf_ splits the nuclear dipole transition via the Zeeman interaction into a sextet of which only the four circularly polarized transitions are excited in this experiment. The four nuclear transitions are excited simultaneously with the same probability by a 50-ps-long broadband x-ray pulse from a synchrotron. The 5-neV linewidth of the ^57^Fe transition leads to coherence times of hundreds of nanoseconds between the transitions such that they form quantum beats over the lifetime of the nucleus of 141 ns. During nuclear resonant forward scattering of synchrotron radiation, primarily one photon at a time interacts with an ensemble of identical nuclei in the thin film to form a collectively excited nuclear state. This nuclear exciton (*31*)—or timed Dicke state (*32*)—decays superradiantly. The control of a collectively excited state is more demanding than that of a single quantum system because the coherences between each of the participating nuclei have to survive when the system is perturbed.

When ferromagnetic magnons are excited resonantly by a radio frequency (RF) magnetic field, the hyperfine field at the nuclei is reduced (*13*) (see Fig. 1A). Because of this reduction, the Zeeman splitting gets smaller, which leads to an energetic shift of the nuclear transitions. In Fig. 1B, the corresponding energy scheme is shown with an energetic shift of the transitions due to the Zeeman interaction and the magnon excitation. The energy shift induced by the magnon preserves the coherences between the nuclear levels (*13*). With magnons present in the ferromagnetic film, the transitions are shifted by ħΔω of a few nanoelectron volts. Exemplarily, the spectrum for a hyperfine field reduction of 3 T is shown. The actual reduction in the hyperfine field is set by the strength of the RF field driving the magnon. During relaxation, the collectively excited nuclei emit a single γ-ray photon within the lifetime of 141 ns, which is in a superposition state of the four allowed dipole transitions each oscillating at its own frequency ω_i_ on the zeptosecond time scale.

**Fig. 2:**
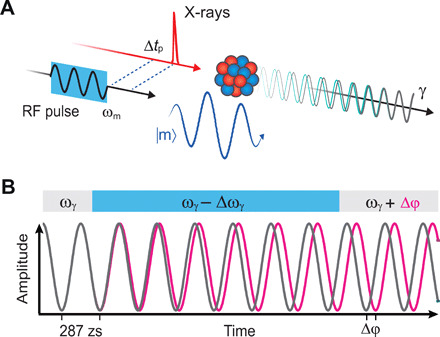
Nuclear dynamics with magnon excitation. (**A**) An ^57^Fe nucleus is excited by an x-ray pulse and subsequently emits a γ-ray photon. The emitted photon is in a superposition state of the four nuclear dipole transitions oscillating at different frequencies, here only shown for two frequencies of same polarization (green and gray). An RF burst with delay Δ*t*_p_ excites the ferromagnetic magnon (blue) and leads to a nanosecond shift of the resonance line as shown in Fig. 1. (**B**) The oscillation of the nuclear transition dipole moment of a single transition at frequency ω_γ_ is shown in gray. During the period indicated by the blue bar, the magnon is excited and the oscillation frequency is shifted by Δω_γ_. Afterward, the oscillation frequency is the same as before, but shifted by Δφ (magenta). This process happens for all four transitions although with different phase shifts.

When the magnon is excited not continuously but by a short nanosecond-long RF burst, the energetic shift in the nuclear dipole transition takes place on the time scale of the transient magnon. In metallic ferromagnets, the magnon frequencies are in the gigahertz range, and their intrinsic magnetic damping times are about 1 ns. In this experiment, magnons are in a classical coherent state also known as a spin wave. The scheme of excitations is depicted in Fig. 2A. Because the phase of the nuclear transition dipole oscillation Δφ(*t*) depends on its momentary frequency ω_γ_ according to φ(*t*) = ∫ω_γ_(*t* ′)*dt*′, any change of this frequency will modify the dynamic quantum phase as schematically depicted in Fig. 2B. By changing the frequency of the nuclear transition dipole moment by Δω_γ_ over the time span of the magnon excitation Δ*t*_m_ indicated by the blue bar, a phase shift Δφ = Δω_γ_Δ*t*_m_ is generated. This phase shift remains after the magnon is damped out. As seen from Fig. 1B, the energetic shifts are different for the inner and outer lines as are the phase shifts. The energetic shifts due to the magnon are on the order of a few nanoelectron volts for the 14.4-keV nuclear transition, and the ratio of the shifts to the nuclear transition energy spans 12 orders of magnitude. To obtain temporal shifts Δ*t*_γ_ = Δφ/ω_γ_ in the oscillation of the nuclear transition dipole moment that are in the zeptosecond range, relative frequency changes have to be applied over the same ratio, resulting in nanosecond transient magnons needed for control. This coincides with the intrinsic nanosecond time scale of the magnon.

**Fig. 3:**
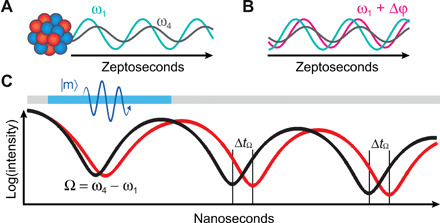
Quantum beat phase shift. (**A**) Schematic phase relation of dipole radiation emitted from the nucleus at transitions 1 and 4 (same polarization) on the zeptosecond time scale. Their interference leads to a quantum beat over nanoseconds [black in (**C**)] at the quantum beat frequency Ω = ω_4_ − ω_1_. (**B**) Phase relation at a later time when a relative zeptosecond phase shift Δφ is generated (magenta) compared with the undisturbed oscillation (gray and green). The red curve in (C) shows the quantum beat resulting from a zeptosecond phase shift Δφ generated by the magnon, which is applied over the nanosecond period indicated by the blue bar. The zeptosecond phase shift is visible as a temporal shift Δ*t*_Ω_ in the quantum beat on the nanosecond time scale.

For an experimental demonstration, we use nuclear resonant forward scattering of x-rays from a ferromagnetic permalloy thin film in which ^57^Fe nuclei are embedded (see Materials and Methods). The magnetic hyperfine field *B*_hf_ is aligned parallel to the wave vector of the incident x-rays in the Faraday geometry in which only the four transitions belonging to the Δ*m* = ±1 transitions can be excited. In the scattered radiation, the two equally spaced lines that belong to the doublets of the transitions with same polarization interfere, resulting in a quantum beat with a single frequency of Ω ≈ 50 MHz (see Fig. 3). Dynamic quantum phase control is achieved through the magnon. Phase shifts in the coupled light-matter state of the nuclear ensemble and the scattered single photon are induced when a few-cycle RF magnetic field burst, synchronized with the incident x-ray pulse, resonantly stimulates the uniform and coherent magnon mode of the ferromagnetic film over a time interval Δ*t*_m_ of a few nanoseconds. During that time, the transient magnon shifts the nuclear transitions as described previously, which results in a phase shift Δφ*_i_* and a corresponding temporal shift Δ*t_i_* of the carrier wave of each of the transitions.

The long coherence times between the nuclear transitions and the formation of quantum beats allow for an interferometric detection of phase changes that are much smaller than the oscillation period of the transitions. The resolution of the phase change is only limited by the temporal resolution of the quantum beat. The zeptosecond temporal shifts Δ*t_i_* of the carrier frequencies ω*_i_* are detected by analyzing the quantum interference between the two resonances of a doublet, e.g., ω_1_ and ω_4_ (see Fig. 3). The temporal quantum beat pattern oscillates at the much smaller frequency Ω = ω_4_ − ω_1_. When the nuclear transitions exhibit a phase shift Δφ*_i_* and a corresponding temporal shift Δ*t_i_* after magnon excitation, the phase shift in the quantum beat is Δϕ = ω_4_Δ*t*_4_ − ω_1_Δ*t*_1_ (see Materials and Methods). Thus, the oscillation frequency of each transition and its temporal shift define the phase shift in the quantum beat (see Fig. 3C). Accordingly, the interferometric detection transforms zeptosecond shifts of the individual nuclear transition dipole moments to nanoseconds shifts in the quantum beat pattern, which allows detection of the magnon-induced zeptosecond temporal shifts.

The energetic shifts of each transition and, hence, their phase shifts are exactly known from the Zeeman interaction. The phase shift for the specific transition Δφ*_i_* is related to the phase shift of the quantum beat Δϕ via

$$\Delta \varphi_{i}=\frac{m_{e}g_{e}-m_{g}g_{g}}{g_{e}-g_{g}}\Delta \phi$$ (1)

where *m*_e_, *m*_g_ are the magnetic quantum numbers and *g*_e_*, g*_g_ are the *g*-factors for the respective transitions between the excited state $|\frac{3}{2};m_{e}\rangle$ and ground state $|\frac{1}{2};m_{g}\rangle$. Thus, we directly determine the nuclear phase shifts Δφ*_i_* from the interferometrically detected phase shift in the quantum beat Δϕ, invoking only the magnetic quantum numbers and *g*-factors of the nuclear states. The relations for the four transitions are Δφ_6,1_ = ±0.86 Δϕ and Δφ_4,3_ = ±0.14 Δϕ. The determination of the carrier phase shift is of same simplicity as relating a temporal delay of a laser pulse to a path difference of a delay line. The determination of the quantum phase or temporal shift in each transition dipole moment is thus possible directly from the quantum beat phase shift.

Flexible phase control is enabled by the excitation parameters of the quasi-particle. This includes the magnon’s driving frequency, its excitation strength, and duration Δ*t*_m_, all set by the parameters of the RF burst. The magnon exhibits a typical Lorentzian response to the driving frequency around its resonance. Thus, both the excitation frequency and amplitude determine the reduction in the magnetic hyperfine field Δ*B*_hf_ and the level splitting (*13*). The relative phase shift of the interacting transitions determines the phase shift of the quantum beat that is given via (see Materials and Methods)

$$\Delta \phi =\Delta \varphi_{j}-\Delta \varphi_{i}=(g_{g}-g_{e})\frac{\mu_{N}}{B_{\text{hf}}}\Delta B_{\text{hf}}\Delta t_{m}$$ (2)

which shows that the phase shift is controlled by the magnon excitation power and its duration.

**Fig. 4:**
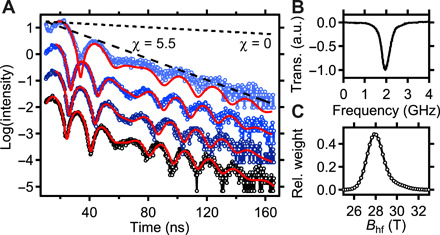
Data for continuous magnon excitation. (**A**) Quantum beat pattern without excitation (black) and excitation powers of 13, 19, and 23 dBm (blue), from bottom to top. Graphs are offset for clarity. The corresponding fits to the nuclear quantum beats are shown in red and result in hyperfine field reductions of −1.5, −2.3, and − 6.5 T, respectively. (**B**) Electrical RF transmission of the stripline showing the ferromagnetic resonance at 1.98 GHz at an external magnetic field of 5 mT, which is continuously excited during the measurements shown in (A). (**C**) Hyperfine field distribution in permalloy, as derived from the data without excitation. a.u., arbitrary units.

Quantum beat patterns with continuous excitation of the magnon at 1.98 GHz are shown in Fig. 4. The nuclei decay superradiantly as can be seen from the accelerated decay shown in Fig. 4A. The speedup parameter χ describes the increased exponential decay of the collectively excited nuclear state (see Materials and Methods). Comparing the speed up of the superradiant nuclear decay χ = 5.5 with the decay of a single nucleus χ = 0 shows that the ensemble of excited nuclei indeed form the nuclear exciton (*33*). The magnon excitation does not perturb the superradiant state, and thus, the magnon preserves the coherence of the nuclear exciton, which is an important prerequisite for coherent quantum phase control of collective quantum states. The reduction in the magnetic hyperfine field induced by the magnon sets the energetic shift Δω*_i_* of the nuclear transitions. From fits of the quantum beats, we determine the hyperfine field distribution centered on 27.9 T (Fig. 4C) and its reduction induced by the magnon, which is up to −6.5 T at an RF power of 23 dBm. For this field reduction, the energetic shift is up to a few tens of nanoelectron volts.

**Fig. 5:**
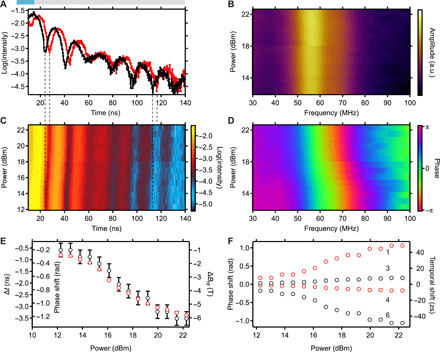
Power dependence of pulsed magnon excitation. (**A**) Quantum beat pattern without excitation (black) and with a pulsed magnon excitation of 22.3 dBm for a duration of 15 ns (red). The blue bar indicates the ontime of the magnon. (**C**) Color plot of the quantum beat pattern in dependence of the magnon excitation power. (**B**) Amplitude and (**D**) phase of the Fourier transform of the quantum beat pattern shown in (C). (**E**) Measured temporal shift and phase shift of the quantum beat and the associated hyperfine field reduction. Red dots show the phase shift determined from the Fourier transform. (**F**) Phase shift and temporal shift of the single transitions, labeled as in Fig. 1, for the right circularly (red) and left circularly (black) polarized transitions.

Figure 5 shows data obtained during transient magnon excitation. Here, the RF pulse is applied before the x-ray pulse arrives, which ensures that the hyperfine field is already reduced when the nuclear exciton is created. The RF pulse is turned off 15 ns after the x-ray pulse, which defines the magnon duration Δ*t*_m_. The comparison of the nonexcited quantum beat pattern with the one excited at 22.3 dBm clearly shows a delay of the quantum beat by Δ*t*_Ω_ = −3.52 ns (dotted lines in Fig. 5, A and C). The delay is stable over time, while the quantum beat pattern is changed only during the 15 ns the magnon is excited, of which only 5 ns is experimentally detectable. The measured quantum beats for various excitation powers, and thus hyperfine field reductions, display a clear change of the phase shift with RF power. From the Fourier transforms of the quantum beat pattern (Fig. 5B), it is evident that the beat frequency Ω centered at 55.8 MHz does not change. The change of the quantum beat frequency on the first 5 ns of the time window is too short to be detectable in the Fourier transform. The phase of the beat pattern, however, is shifted considerably with pumping power (see Fig. 5D) because the spin precession angle of the magnon and the associated reduction in the hyperfine field increase (*13*). The temporal and phase shifts of the quantum beat are plotted in Fig. 5E together with the hyperfine field reduction determined from Eq. 2. For comparison, the phase shift of the Fourier transform is also shown. The phase and zeptosecond temporal shifts of the single transitions are plotted in Fig. 5F. Because of the change of the Zeeman splitting by the magnon, the frequency of the transitions either increases or decreases. This defines the sign of each transition’s phase shift. The smallest detectable shift is 1.3 zs. Taking the timing stability of our setup into account in the model (see Materials and Methods), we can calculate the timing stability of the temporal shift to below 50 ys.

**Fig. 6:**
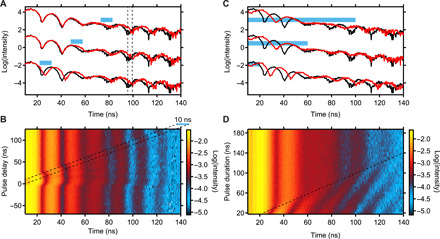
Timing dependence of pulsed magnon excitation at 22.3 dBm. (**A** and **B**) Quantum beat patterns in dependence of the RF pulse delay between a 10-ns transient magnon and the nuclear excitation. The lines in (A) indicate the induced temporal shift after magnon excitation. Graphs are offset for clarity. In (B), the dashed lines indicate the 10-ns magnon duration and its relative timing to the x-ray pulse at time zero. For positive delays, the magnon is excited after the nuclear exciton. (**C** and **D**) Quantum beat patterns in dependence of the pulse duration that excites the magnon. In (C), blue bars exemplarily indicate the ontime of the magnon. Graphs are offset for clarity. In (D), the magnon is already excited when the nuclear exciton is triggered. The dashed line indicates when the magnon excitation stops.

To further verify our model, the dependence of the quantum beat on the magnon timing and duration is measured. At constant excitation power, the magnitude of the phase shift predicted by our model (Eq. 2) depends on the magnon duration only and is independent of the moment the magnon is initiated. The dependence on the delay Δ*t*_p_ is shown in Fig. 6 (A and B). In Fig. 6A, the quantum beats for three different delays of the magnon are shown. Here, the magnon is excited after the nuclear exciton. The phase shift of the quantum beat is constant regardless of the moment in time when the magnon alters the level splitting. Before the magnon is driven, the nuclei are in the same quantum state as seen from the quantum beat patterns that are all identical in the region of the unperturbed quantum beat before the pulse arrives. After magnon excitation, the nuclei show the same quantum beat pattern for all delays. This beat pattern is the same as for the undisturbed quantum beat but incorporates the dynamic quantum phase shift. Thus, the nuclear exciton returns to its initial quantum state after the perturbation by the magnon but with the imprinted quantum phase. In addition, the coherence properties of the nuclear exciton are preserved for the transient magnon excitation. This must be the case because otherwise incoherent damping of the quantum beats with increasing time after excitation would have been observed (*34*). In Fig. 6B, the whole dependence on the magnon delay is shown and the linear dependence is clearly visible. In addition, for pulse delays less than −10 ns, the magnon does not influence the quantum beat as it is already damped out before the nuclear exciton is created.

In Fig. 6 (C and D), the phase dependence on the magnon duration Δ*t*_m_ is shown. Here, the magnon was applied before and during x-ray excitation. In this case, the level scheme with smaller Zeeman splitting is present. The nuclei pick up a positive phase shift when the magnon is turned off. For the longer magnon durations used, it is clear that the quantum beat frequency is reduced compared with the undisturbed one, best visible for the longest pulse lengths in Fig. 6 (C and D). It is observed that the induced phase shift due to magnon excitation increases linearly with magnon duration. At magnon duration of 60 ns, a phase change of approximately −2π is visible in the data. Our analytical theory in the kinematical approximation predicts a phase change of −1.7π. This is close to the phase change visible in the data but does not account for dynamical x-ray scattering effects. These effects disturb the quantum beat at later times and are best visible between 60 and 100 ns. The hyperfine field distribution does not play a role in the observed temporal shift (see Materials and Methods).

## DISCUSSION

The experimental data are in perfect accordance with the model and demonstrate that the collective nuclear quantum state can be coherently controlled with magnons. A high-energy resonance can be manipulated on an energy scale that is nine orders of magnitude smaller than its kiloelectron volt transition energy, i.e., with magnons of microelectron volt energy. The magnon is used to tune the nuclear-level scheme of the transition on its intrinsic lifetime. Thereby, the transient magnon allows one to shift the dynamic quantum phase of the nuclear dipole transitions and of the single γ-ray photon emitted by the nuclei. This control scheme shifts the quantum phase with zeptosecond precision and a timing stability of 50 ys. So far, the study and control of quantum phases and time delays have been limited to the attosecond time scale so that access to the zeptosecond changes were not possible. In our experiment, the quantum phase shift is resolvable down to 1 zs, corresponding to one natural unit of time ħ/(*m*_e_c^2^) = 1.29 zs (*35*).

Because of the preservation of the coherence of the collective nuclear state, our approach can be used to establish efficient coherent control schemes, for example, to precisely time the emission of hard x-rays, to tune nuclear clock transitions (*36*), or to advance spectroscopic techniques (*7*). The transient magnon excitation is a sudden switch between two different level configurations where the quantum information of the states is stored in the phase shift of a single-photon state. More advanced schemes may be envisioned such as using double pulses or different envelopes to tune the carrier envelope of the phase. This carrier envelope phase tuning is important in nonlinear light-matter interactions (*37*) but was not possible at x-ray energies so far.

Although the phase control is demonstrated on a collective quantum state, the quasi-particle control scheme could also be used to tune single quantum systems. Other quasi-particles of condensed matter, like phonons, plasmons in conductors, excitons in insulators or semiconductors, or Cooper pairs in superconductors, are possible testbeds for tuning specific interactions of the solid to the embedded quantum system. The actual energies of the quasi-particle and its excitation process by microwave, terahertz, or light pulses, should not be important for these schemes as long as the quasi-particle modifies the embedded quantum state during the quasi-particle’s excitation time and lifetime. In our case, the magnon excitation time is comparable to the lifetime of the nuclear state. However, other scenarios are possible with much different lifetimes depending on the interaction strength of both constituents. Moreover, because of their largely different energies, the magnon oscillation in this experiment is not coherent to the nuclear dipole oscillation nor the x-ray pulse, although its excitation time is synchronized to the x-ray pulse. For quasi-particles and quantum systems that have similar energies, a coherent excitation among them is possible and might bring an additional control handle on the quantum system. In general, we have demonstrated that zeptosecond single-photon interferometry using electromagnetically induced excitations of quasi-particles is a promising tool for coherent dynamic control of atomic or nuclear quantum states in solid-state environments.

## MATERIALS AND METHODS

### Sample preparation

Samples are prepared by electron beam lithography, lift-off processing, and sputter deposition. A 10-μm-wide gold stripline is prepared on a GaAs wafer (*13*). On top of the stripline, a much wider trilayer (Ta 15 nm/Ni_80_^57^Fe_20_ 13 nm/Ta 3 nm) is deposited in which the iron is enriched to 95% in the isotope ^57^Fe. The trilayer is electrically insulated from the gold stripline by a hydrogen silsesquioxane (HSQ) film that also flattens the surface of the stripline. All parts of the ferromagnetic film that do not overlap the stripline are covered with an additional gold layer, such that only the excited part of the ferromagnetic film contributes to the nuclear reflectivity.

### Nuclear resonant scattering

Nuclear resonant scattering has been performed at the Dynamics beamline P01 at the synchrotron radiation source PETRA III, DESY, Hamburg. The energy of the x-rays is tuned to the 14.4-keV resonance of ^57^Fe. The nuclei’s number density is 1.8 × 10^28^ m^−3^ and such that a nuclear exciton is formed when the 14.4-keV nuclear resonance of the isotope is excited by an x-ray pulse from the synchrotron. Measurements are performed in Faraday geometry, where the x-ray wave vector is parallel to the static in-plane magnetization of the film set by the external magnetic field. The x-rays are monochromatized by a high-heat load monochromator and a high-resolution monochromator to a bandwidth of about 1 meV. Two Kirkpatrick-Baez mirrors focus the x-rays down to a spot size of 10 μm × 5.7 μm (horizontal × vertical) on the stripline. Measurements are performed in grazing incidence at the critical angle of the trilayer at 0.27°, resulting in a length of the x-ray footprint of 1.2 mm on the sample. The synchrotron was operated in 40 bunch mode with an x-ray repetition rate of 5.2 MHz. A time-gated avalanche photo diode (APD) counts the delayed single photons from the nuclear de-excitation in a time-resolved manner to measure the nuclear quantum beat. The measured intensity shown is the number of counts per time interval divided by the total counts per quantum beat pattern. The measurements start 10 ns after nuclear excitation due to the electronic downtime of the detector after the prompt pulse.

### Magnon excitation

A vector network analyzer is used to measure the RF absorption and to detect the magnon resonance. It also serves as the source for the high-frequency continuous wave (CW) signal with a frequency *f* = 1.98 GHz to excite the magnon resonantly. The magnon resonance frequency is tuned to the excitation frequency *f* via a static magnetic field *H*_ext_. The static magnetic field aligns the static magnetization along the stripline’s long axis, which coincides with the *k*-vector of the incoming x-rays. The dynamic magnetic Oersted field of the stripline is thus perpendicular to the static magnetization and excites the ferromagnetic mode (*k* = 0) of magnons directly above the stripline. To perform timing experiments, a pulse generator with variable pulse duration and delay time Δ*t*_p_ is triggered by the bunch clock of the synchrotron at a frequency of 5.2 MHz. The CW signal and the pulses are multiplied in a mixer and subsequently amplified. Samples are contacted by RF probes. The stripline generates a magnetic Oersted field in the permalloy film with the same time structure as the electrical current (*13*). The resulting magnetic field burst is resonant to the ferromagnetic magnon in the permalloy film. The time delay Δ*t*_p_ between the x-ray pulse and the burst can be set freely within the x-ray bunch repetition period of the synchrotron of 192 ns.

### Quantum phase shift

The transition dipole moment of a nucleus after excitation is

$$A(t)=A_{0}\theta (t)\text{exp}(-\frac{t}{2\tau_{0}}-i\varphi (t))$$ (S1)

with the phase factor $\varphi (t)=\int_{0}^{t}\omega_{\gamma }(t\prime )\mathrm{dt}\prime$, where Γ_0_ = 4.66 neV is the natural linewidth of the resonance connected to the decay rate via τ_0_ = ħ/Γ_0_ = 141 ns. For an undisturbed system, we find φ(*t*) = ω*_i_t*, where ω*_i_* is the resonance frequency of the *i*-th transition within the sextet of the hyperfine-split nuclear resonance of ^57^Fe. The phase is tuned by a transient temporal change of ω*_i_*(*t*).

Our magnonic system is assumed to change slowly on the time scale of the nuclear dipole transition given by the nuclear period of 287 zs. In this case, the system is in an instantaneous eigenstate of the Hamiltonian at any point in time. Thus, the transition energy is always the eigenvalue of the momentary Hamiltonian

$$E_{i}+\Delta E_{i}=\hbar (\omega_{i}+\Delta \omega_{i})=E_{i}-(m_{e}g_{e}-m_{g}g_{g})\mu_{N}\Delta B_{\text{hf}}(t)$$ (S2)

which gives the energetic shift of the *i*-th transition *E_i_* = *E*_0_ − (*m*_e_*g*_e_ − *m*_g_*g*_g_)μ*_N_B*_hf_ in time due to the magnetic hyperfine field reduction Δ*B*_hf_ upon magnon excitation. The *g*-factors for the ground and excited nuclear states are *g*_g_ = 0.18124 and *g*_e_ = −0.102, respectively.

In the case of σ-polarized synchrotron radiation and no polarization analysis in the detection process, we obtain for the time-dependent (delayed) scattered intensity in reflection geometry (*33*)

$$I(t)\approx ({|{\tilde{f}}_{\sigma \sigma }|}^{2}+{|{\tilde{f}}_{\sigma \pi }|}^{2})\chi^{2}e^{-\chi t/\tau_{0}}$$ (S3)

with the Fourier transform of the scattering matrix $\tilde{f}(\omega )$ describing nuclear scattering and the speedup parameter χ accounting for the excitonic nuclear decay. In the Faraday geometry, the scattering matrix elements depend on the circular polarized nuclear resonant scattering functions *F*_−1_ and *F*_+1_ only, and the transitions ω_2_ and ω_5_ with Δ*m* = 0 cannot be excited. The intensity is given by

$$\begin{array}{c} I(t)∼(2{|{\tilde{F}}_{+1}|}^{2}+2{|{\tilde{F}}_{-1}|}^{2})\chi^{2}e^{-\chi \frac{t}{\tau_{0}}}\\ ∼(\frac{10}{3}+\text{cos}(\varphi_{4}(t)-\varphi_{1}(t))+\text{cos}(\varphi_{6}(t)-\varphi_{3}(t)))\chi^{2}e^{-(1+\chi )\frac{t}{\tau_{0}}} \end{array}$$ (S4)

with the phase factors of the specific transition φ*_i_*. The energy difference of the two lines belonging to the right-circular (Δ*m* = −1) and left-circular (Δ*m* = 1) transitions defines the quantum beat frequency Ω. Without the phase shift, we find φ_4_(*t*) − φ_1_(*t*) = φ_6_(*t*) − φ_3_(*t*) = (ω_4_ − ω_1_)*t* = Ω*t*. This means that only one beat frequency is present.

The duration of the magnon excitation is modeled by a Boxcar function ∏_*t*_s_, Δ*t*_m__(*t*) = Θ(*t* − *t*_s_) − Θ(*t* − *t*_s_ − Δ*t*_m_), where *t_s_* is the start time of the magnon and Δ*t*_m_ is its duration. After magnon excitation, *t* > *t_s_* + Δ*t*_m_, the phase is φ*_i_*(*t*) *=* ω*_i_t +* Δφ*_i_* with the phase shift Δφ_i_ = Δω*_i_*Δ*t*_m_. The associated temporal shift in the transition dipole moment is

$$\Delta t_{i}=\frac{\Delta \varphi_{i}}{\omega_{i}}=\frac{\Delta \omega_{i}}{\omega_{i}}\Delta t_{m}$$ (S5)

From this model, we find for the relative phases of Eq. S4 after magnon excitation

$$\text{cos}(\varphi_{4}(t)-\varphi_{1}(t))+\text{cos}(\varphi_{6}(t)-\varphi_{3}(t))=2\text{cos}(\Omega t+\Delta \phi )$$ (S6)

where the measured quantum beat phase shift is given by the relative phase shift of the transitions Δϕ= Δφ_4_ − Δφ_1_ = Δφ_6_ − Δφ_3_. Then, the measured intensity of the quantum beat is

$$I(t)∼(\frac{5}{3}+\text{cos}(\Omega t+\Delta \phi ))\chi^{2}e^{-(1+\chi )\frac{t}{\tau_{0}}}$$ (S7)

For the determination of the phase shift Δϕ in the quantum beat and the corresponding delay Δ*t*_Ω_, we use Eqs. S2 and S6 as well as the undisturbed beat frequency Ω to obtain Eq. 2 and

$$\Delta t_{\Omega }=\frac{\Delta \phi }{\Omega }=\frac{g_{g}-g_{e}}{\hbar \Omega }\mu_{N}\Delta B_{\text{hf}}\Delta t_{m}=\frac{\Delta B_{\text{hf}}}{B_{\text{hf}}}\Delta t_{m}$$ (S8)

As the phase shifts depend on the duration of the magnon Δ*t*_m_ as Δφ*_i_* = Δω*_i_*Δ*t*_m_ and Δϕ = ΔΩΔ*t*_m_, the phase shifts found in Eq. 2 have the same ratio as the energetic shifts of the quantum beat and the individual transitions, leading to ℏΩ = ℏ(Δω_j_ − Δω_j_). With Eqs. 2 and S2, we find $\left(g_{e}-g_{g})=(m_{e}^{j}g_{e}-m_{g}^{j}g_{g})-(m_{e}^{i}g_{e}-m_{g}^{i}g_{g}\right)$, which leads to elementary Eq. 1.

### Timing stability of the phase shift

An important quantity is the stability of the induced phase and temporal shift. The phase and temporal resolution of the experiment is given by the time resolution of the quantum beat, which is limited by the APD to about 0.5 ns. The smallest detectable temporal shift in each transition is on the order of a zeptosecond. The temporal stability, however, is much better. Small uncertainties of material parameters, like the dynamic magnetic susceptibility and *g*-factors and so on, do not enter the timing stability as these values are constants. The temporal stability is given by the errors in the magnon excitation only. This incorporates the excitation power and the duration distribution of the frequency burst that is applied.

The RF generator has a very precise power output of ~0.05 dB (~0.01%) and frequency output precision of ±2 parts per million (ppm). The power error can be neglected compared with the precision of the pulse generator whose signal is mixed with CW from the RF generator. The magnon resonance width is 300 MHz, and the ppm error on the 2-GHz excitation frequency of the RF generator can be neglected.

The main errors come from the voltage pulse that is mixed with the CW signal to generate the frequency burst. It has a voltage level accuracy of ~1.5% and a timing precision of ~2%. Note that these values as given by the data sheet do not give the stability itself but the reproducibility when changing the setup. Although the stability is expected to be better, we take these values as the upper error boundary for the timing stability.

The temporal shift is given by

$$∆t_{i}=\frac{∆\omega_{i}}{\omega_{i}}∆t_{m}=-\frac{m_{e}g_{e}-m_{g}g_{g}}{E_{i}}\mu_{N}∆B_{\text{hf}}∆t_{m}$$ (S9)

The magnetic field reduction ∆*B*_hf_ depends on the opening angle of the magnetization precession angle ϑ as

$$∆B_{\text{hf}}=B_{\text{hf}}(\text{cos}(\eta \vartheta )-1)$$ (S10)

In a thin film, magnetization dynamics is highly elliptical, and in our thin film geometry, the dynamic out-of-plane magnetization component is about a factor of 13 smaller than the in-plane component ϑ. The factor η = 0.538 accounts for this ellipticity of the magnetization precession (*13*), and the average precession angle is ηϑ. In total, we arrive at

$$∆t_{i}=-\frac{m_{e}g_{e}-m_{g}g_{g}}{E_{i}}\mu_{N}B_{\text{hf}}(\text{cos}(\eta \vartheta )-1)∆t_{m}$$ (S11)

In the linear regime of the magnon excitation, which is just about satisfied for the small excitation powers where the smallest shifts are detected, the magnon opening angle is proportional to the magnetic excitation field, which is proportional to the applied voltage. Despite the ellipticity and actual precession angles, the effective model on how the magnetization precession cone affects the hyperfine field is almost linear in this regime (*13*). Therefore, the inaccuracy of the in-plane precession angle ϑ is to first-order proportional to the one of the voltage pulse. Using propagation of uncertainties, we obtain for the uncertainty δ*t_i_* of the temporal shift ∆*t_i_*

$$\delta t_{i}=\sqrt{{\left(-\frac{m_{e}g_{e}-m_{g}g_{g}}{E_{i}}\mu_{N}B_{\text{hf}}(\text{cos}(\eta \vartheta )-1)\delta t_{m}\right)}^{2}+{\left(\frac{m_{e}g_{e}-m_{g}g_{g}}{E_{i}}\mu_{N}{\eta B}_{\text{hf}}\text{sin}(\eta \vartheta )∆t_{m}\delta \vartheta \right)}^{2}}$$ (S12)

With the values from the experiment of *B*_hf_ = 28 T, ∆*t_m_* = 15 ns, ϑ = 0.498 rad (ηa = 14.3°, as calculated from Eq. S10 for the smallest detectable phase shift of 1.3 zs), and the appropriate errors δ*t*_m_, δ given above, one arrives at the smallest timing uncertainty for transitions 3 and 4 of δ*t*_3,4_ = 47 ys. Note that this value is an upper boundary, and the real timing uncertainty is expected to be smaller. With even more precise pulse generators, this value can be further reduced.

### Hyperfine field distribution

In permalloy, the hyperfine field distribution ranges from 26 to 31 T with a peak at about 28 T. The accelerated decay of the nuclear exciton cannot be explained by the width of the hyperfine field distribution, which contributes only marginally to the decay rate. The quantum phase shift will be different for various hyperfine field values due to different reductions Δ*B*_hf_ for each hyperfine field value. Thus, the transition dipole oscillation frequencies will also differ. However, this does not influence the temporal shift measured in the quantum beat. From the model of the hyperfine field reduction by the magnon (*13*), we conclude that the influence of the magnon is a relative reduction in the hyperfine field that scales with the precession angle of the magnetization, which is uniform over the sample. The ratio Δ*B*_hf_/*B*_hf_ in Eq. S8 leads to a temporal shift that is the same for every hyperfine field. Thus, the hyperfine field distribution does not influence the time delay. The different beat frequencies encountered for the various hyperfine field values normalize the different phase shifts to the same temporal shift in the quantum beat. This is the reason why no blurring effects are observed due to the hyperfine field distribution in the phase-shifted quantum beats.
